# A historical perspective of malaria policy and control in India

**DOI:** 10.1016/j.ijregi.2024.100428

**Published:** 2024-08-21

**Authors:** Avik Kumar Sam, Siddhartha Karmakar, Siuli Mukhopadhyay, Harish C. Phuleria

**Affiliations:** a Environmental Science and Engineering Department, Indian Institute of Technology Bombay, Mumbai, India; b Department of Mathematics, Indian Institute of Technology Bombay, Mumbai, India; c Inter Disciplinary Program in Climate Studies, Indian Institute of Technology Bombay, Mumbai, India; d National Disease Modelling Consortium, Indian Institute of Technology Bombay, Mumbai, India

**Keywords:** Malaria, Policy, Review, Climate change

## Abstract

Malaria is a major public health problem in India, with ∼0.18 million cases reported in 2022 and 1,309 million population living at risk of infection. The Indian government has introduced various intervention strategies to reduce and manage the number of outbreaks with proper mechanisms. In this policy paper, we have extensively reviewed these intervention strategies using a quantitative approach. The success of the Urban Malaria Scheme, focusing on the 131 urban regions distributed across the country, along with the Intensified Malaria Control Project implemented in the seven hotspots, has been highlighted. The recently formulated National Framework for Malaria Elimination in India has resulted in declining malaria cases in hotspots like Odisha. However, states like West Bengal, Maharashtra, and Tripura have emerged as new hotspots in recent years. A systematic platform for data dissemination and active public-private partnership will expedite malaria elimination in India.

## Highlights

• In India, malaria policies and their targets and outcomes are quantitatively reviewed.
• Insecticidal and drug resistance are the underlying drivers of the interventions.
• Climate change, migration, and healthcare accessibility are the new challenges.
• Increased collaboration between academia, industry, and government is recommended.

## Graphical abstract

**Figure:**
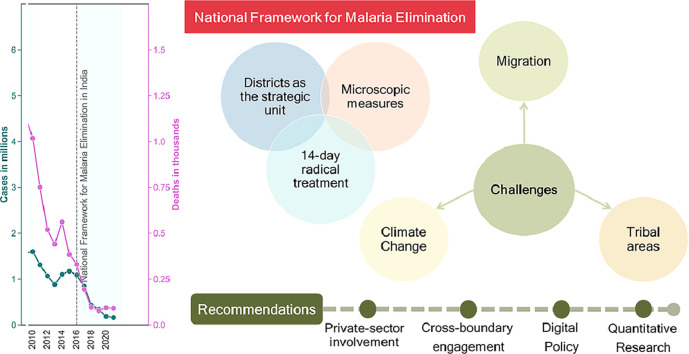

## Introduction to the malaria triangle in India

The epidemiology of malaria in India is complex owing to its geo-ecological and socio-economic diversity as well as the varied distribution of the mosquito vector species. A total of nine species of anopheline mosquitoes are responsible for the transmission of four human plasmodial species – *Plasmodium falciparum, P. vivax, P. malariae, and P. ovale*. Since 2019, *P. falciparum* has been the dominant parasite, accounting for 63.3% of cases in 2020, which decreased to 62.8% and 57.4% in 2021 and 2022, respectively.

**Figure 1:**
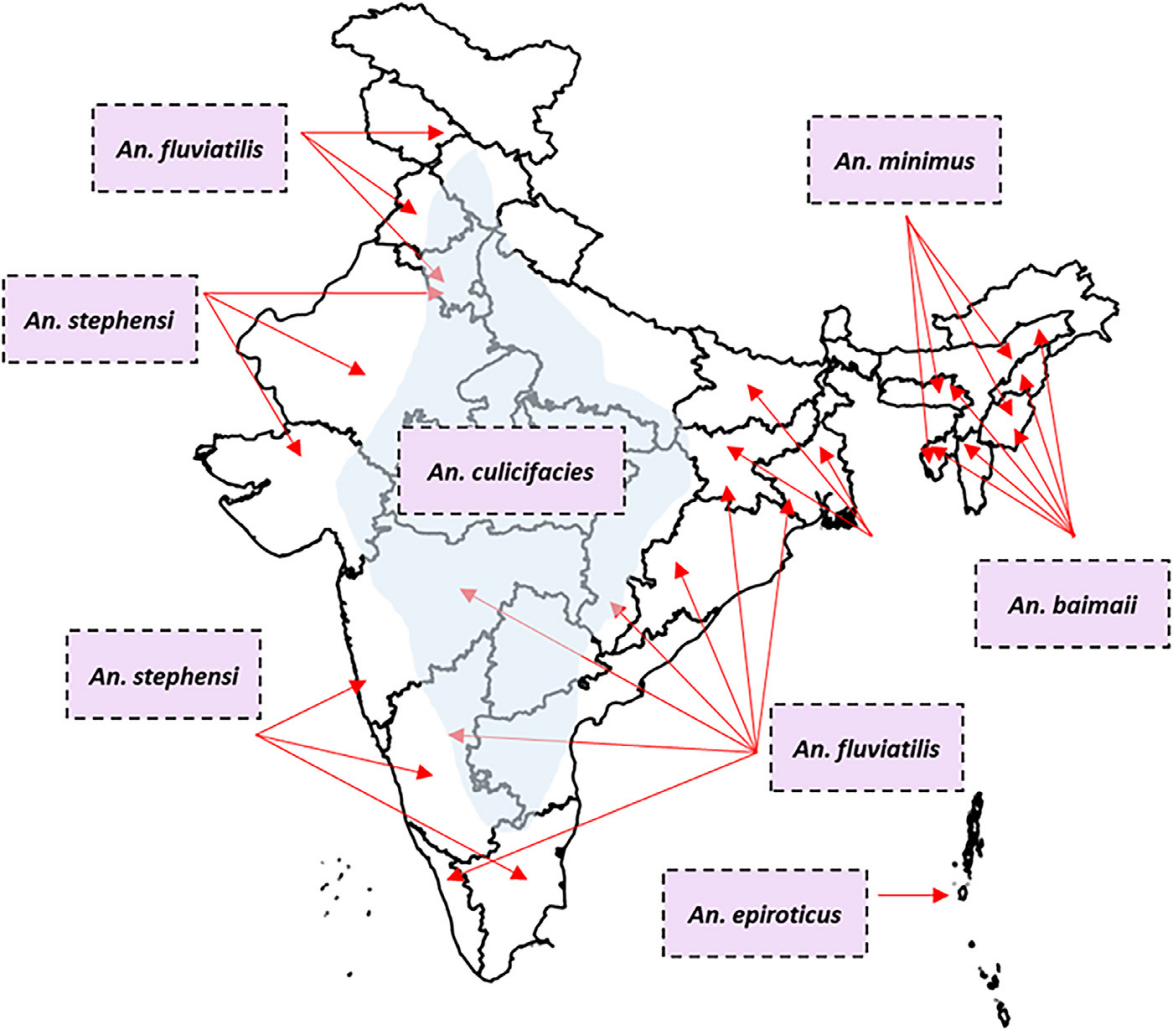
Spatial distribution of the major malaria vectors across India. The distribution data was obtained from existing literature and government reports and replotted using Geopandas in Python 3.10.6. The shaded region represents the distribution of the primary vector of malaria transmission, that is, *An. culicifacies*.

Among the mosquito species, *Anopheles culicifacies* is considered the principal vector of malaria in rural regions, responsible for 60-70% of malaria cases annually, while *An. stephansi* is responsible for the transmission in urban areas. *An. minimus* and *An. baimaii* are restricted to the northeastern (NE) regions of India, *An. fluviatilis* is reportedly found in the foothills and plains [1,2]. *An. epiroticus*, preferring brackish water, is an important vector in the Andaman and Nicobar Islands [3]. Figure 1 illustrates the distribution of the vectors responsible for malaria transmission.

When India attained independence in 1947, about 75 million malaria cases and 0.8 million deaths were reported [4]. The Government of India launched the National Malaria Control Program in 1953 as it prioritized healthcare throughout the country. Five years later, the National Malaria Eradication Program (NMEP) was formulated in 1958, which primarily focused on spraying dichlorodiphenyltrichloroethane (DDT) [5]. During the “DDT eradication period” under the NMEP, a reliable surveillance program was developed, leading to higher effectiveness of the program in reducing malaria cases and deaths [6] as zero deaths were reported in 1961, and cases decreased to less than 50,000. In this policy paper, we systematically analyze the effectiveness of policy implementations focusing on malaria control over the years. The role of the World Health Organization (WHO) in supporting national efforts has been reviewed, while the future of malaria control in India is also discussed. Raw data on reported malaria cases and deaths was obtained from the National Center for Vector Borne Disease Control, NCVBDC (formerly NVBDCP), the umbrella organization under the Directorate General of Health Services, Ministry of Health and Family Welfare, Government of India, and reanalyzed for our policy paper.

## Rising resistance to insecticides

The success of the malaria control efforts could not be sustained over the long term, as India reported a post-eradication peak of 6.47 million cases in 1976 [7]. This drastic resurgence was attributed to the increasing insecticidal resistance, rapid urbanization, development projects, migration, and other technical, administrative, and financial operational challenges [[5], [6], [7]]. The irregular and interrupted supply of DDT to the administrative units that were originally designed to cover a population of 1 million emerged as a critical factor in the malaria outbreaks during the 1980s. Furthermore, the initial planning of the NMEP focused on the rural areas and excluded the urban ones. The malaria outbreaks from *An. stephansi* and *An. culicifacies* in the urban areas became more frequent as the urban population increased. The surveillance necessary to eliminate the residual malaria cases was also inadequate due to staff shortages and improper training and supervision [5].

In response to the growing resistance of *An. culicifacies* to DDT and benzene hexachloride across India, the focus for malaria control was shifted to using malathion. Widespread active participation of the community was sought through drug distributions, medical treatment facilities, health workers, local governance councils, and education through schools and volunteers. The impressive success in malaria control during the 1970s led to the apparent belief that malaria was declining in India. The research was deemphasized, evident from the conversion of the Malaria Institute of India into the National Institute of Communicable Diseases. This resulted in a lack of comprehensive documentation on the vulnerability and receptivity of different regions across the country. The control program was reshaped into eradication to reduce costs and eliminate the disease before the emergence of large-scale insecticide resistance. Due to the withdrawal of active malaria control efforts and indifferent approaches, focal outbreaks and severe high incidences were reported in various regions across the country [5,8].

In 1982, a national antimalarial drug policy was implemented to improve case management. Sulfa-pyrimethamine was used to treat *P. falciparum*, specifically in regions where resistance to chloroquine was documented. The efficacy of the drugs was monitored, which served as the basis for formulating treatment strategies and updating policies in the later years. For instance, in 2005, a formulation of artesunate plus sulfadoxine-pyrimethamine was suggested as the first-line treatment in regions with identified drug resistance and second-line treatment in regions where chloroquine treatment failures were reported. This drug became the first-line treatment for the whole country in 2010 [6]. However, a policy mandate in 2013 recommended the use of artemether-lumefantrine in the seven NE states, namely Arunachal Pradesh, Assam, Manipur, Meghalaya, Mizoram, Nagaland and Tripura, due to the growing resistance to artesunate [9].

While indoor residual spraying was the backbone of malaria control, the NMEP in 1985 mandated the application of malariol, Baytex, temephos, and larvivorous fishes. Reduction at source using environmental-friendly biocides like *Bacillus thuringiensis israelensis* and *B. sphaericus* were advocated in areas as a substitute for chemical larvicides. Personal protection through insecticide-treated nets (ITNs) was proposed [10]. The benefits of ITNs on parasite prevalence, mortality, and Entomological Inoculation Rate, an index measuring the intensity of malaria transmission, were documented in Africa, and the cost-effectiveness and the long-lasting effects of the ITNs were highlighted [11]. These ITNs were used in places across India, providing temporary shelters, for instance, to workers in dams, therefore, preventing the possibility of explosive malaria outbreaks. However, the disproportionate usage of ITNs due to the misuse and attitude of the population decreased the overall effectiveness of malaria control [6,12].

Replacement insecticides like malathion, carbamates, dieldrin/hexachlorocyclohexane (HCH), and other synthetic pyrenoids were actively used as the insecticidal approach became the primary choice for malaria control [12]. HCH was forbidden in India from 1997 due to environmental concerns, while DDT was being selectively used for malaria, and kala-azar was followed. The prolonged usage of the insecticides within a geographical region was responsible for the growing resistance, which could potentially be reduced through a rotation policy, as observed in Mexico [13]. It was asserted that effective and continuous monitoring of the resistance status using standardized bioassay techniques provided by the WHO in India was the 'key' to managing insecticide resistance [12]. By 2008, two regions in India witnessed quadruple resistance, while several parts of central India depicted triple resistance, according to the NCVBDC (unpublished). The geographical map of insecticidal resistance across the country is constantly changing and needs to be closely monitored.

## Urban malaria

Urban malaria contributed to the increasing malaria outbreaks in the 1970s, which was neglected during the initial planning of eradication programs, as discussed earlier. The problem became evident when a study in Tamil Nadu revealed that the urban areas accounted for about 80% of the cases between 1964 and 1967 [5]. In response, the Urban Malaria Scheme (UMS) was implemented during 1971-1972. Initially envisaged in 131 towns across 19 states and union territories (UT), the UMS was estimated to protect 142.9 million people. In this scheme, the parasite control focused on primary treatment through passive agencies involving hospitals, public and private dispensaries, and private doctors. Vector control measures comprised reduction at source, usage of larvicides and larvivores fish like *Gambusia,* and employing space spray and minor engineering tools along with adequate legislative measures [7,14]. In 1977, additionally, the Modified Plan of Operations (MPO) was designed to curb malaria in both rural and urban areas through anti-parasitic and anti-larval measures to abate transmission. However, MPO failed to achieve its target, and the National Malaria Control Program was subsequently integrated with the primary health care delivery system [4].

Later in 1997, the Enhanced Malaria Control Project was launched to combat malaria in high-transmission areas with additional World Bank support [14]. The Intensified Malaria Control Project (IMCP) was established with the help of the Global Fund to fight AIDS, tuberculosis, and malaria in 2005. The IMCP aimed for control in 94 districts belonging to 10 states—seven of them in the N/E region through the introduction of rapid diagnostic tests (RDTs) [7,14]. For instance, in Udalgiri, Assam, a significant decline in cases was attributed to interventions including RDTs, long-lasting insecticidal nets, artemether-lumefantrine drugs, and awareness outreaches, as the API declined from 14.9 in 2005 to 2.6 in 2008, showcasing the effectiveness of the IMCP strategies [15].

**Figure 2:**
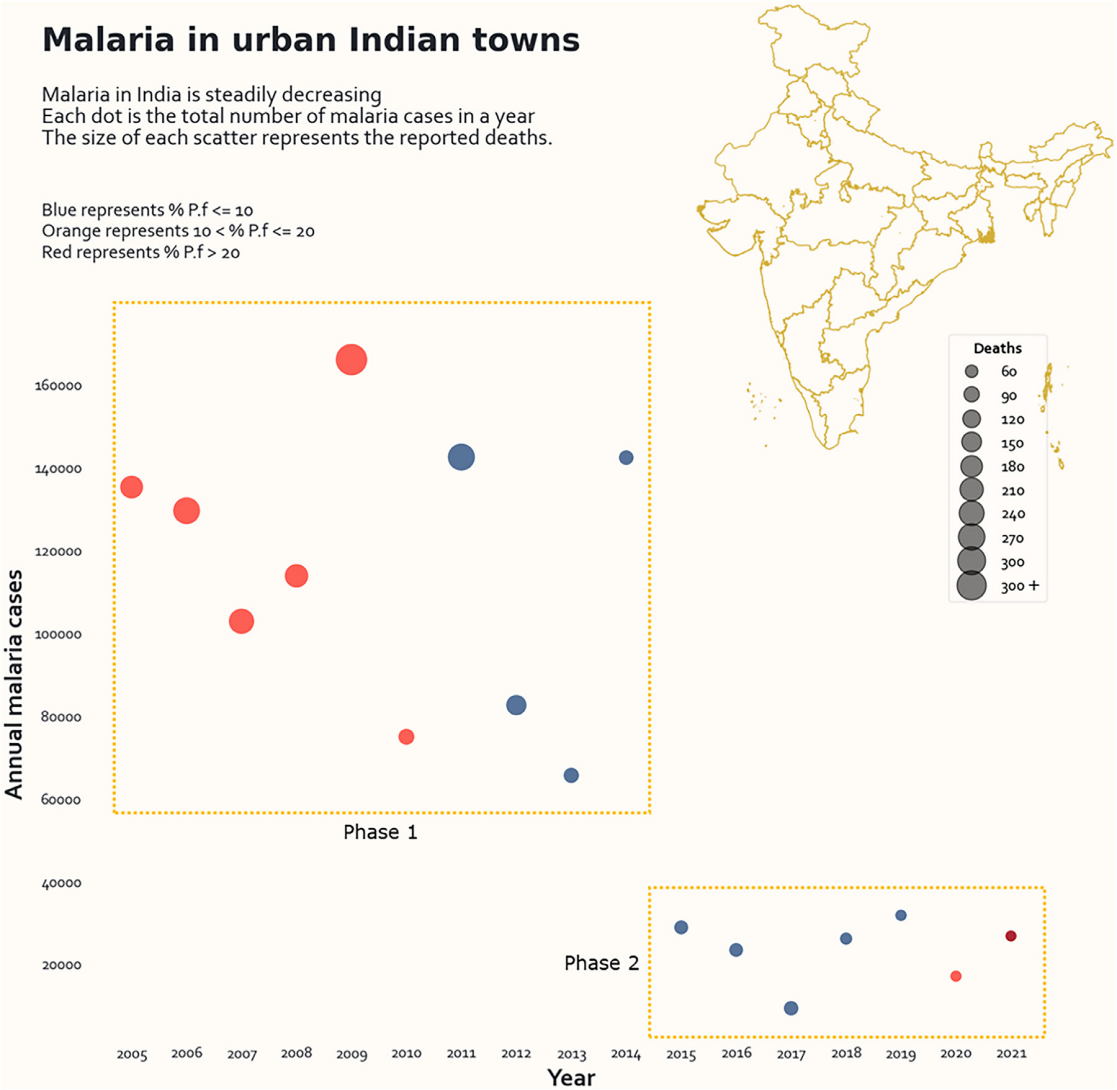
Malaria in urban India is covered under the Urban Malaria Scheme. % P.f indicates the percentage of P. falciparum (Data Source: NCVBDC, unpublished). P.f., P. falciparum.

To ascertain the importance of malaria control in urban areas and the rest of the country, we statistically compared malaria cases and deaths reported between 2007 and 2021. The epidemiological profile of malaria in towns covered under UMS since 2005 is provided in Figure 2. The period between 2005 and 2021 is divided into two distinct phases: phase 1 between 2005 and 2014 and phase 2 between 2015 and 2021. The cases in phase 2 (23,301.7 ± 7711.7) are lower than those observed in phase 1 (115,539.2 ± 33,308.1). The total cases in phase 2 were restricted to <32,000 cases, with a minimum of 9292 reported in 2017, a year after the National Framework for Malaria Elimination in India (NFME) was implemented nationwide. In contrast, the case distribution in phase 1 was highly variable, with three peaks distinctly observed in 2009 (166065), 2011 (142502), and 2014 (142376). Furthermore, the proportion of *P. falciparum* cases in phase 1 varied between 5.1-18.8%, with the maximum reported in 2009 when the reported deaths were also the highest (213).

The reported cases in UMS towns decreased by ∼80% in 2015 compared to 2014, although the reported deaths were very low in both years. The slide positivity rate, defined as the laboratory-confirmed cases in 100 suspected cases, also dropped from 1 to 0.38 in 2015. The drop in malaria cases was ∼55% and ∼42% in 2010 and 2012 when compared to 2009 and 2011, respectively (Figure 2).

**Figure 3:**
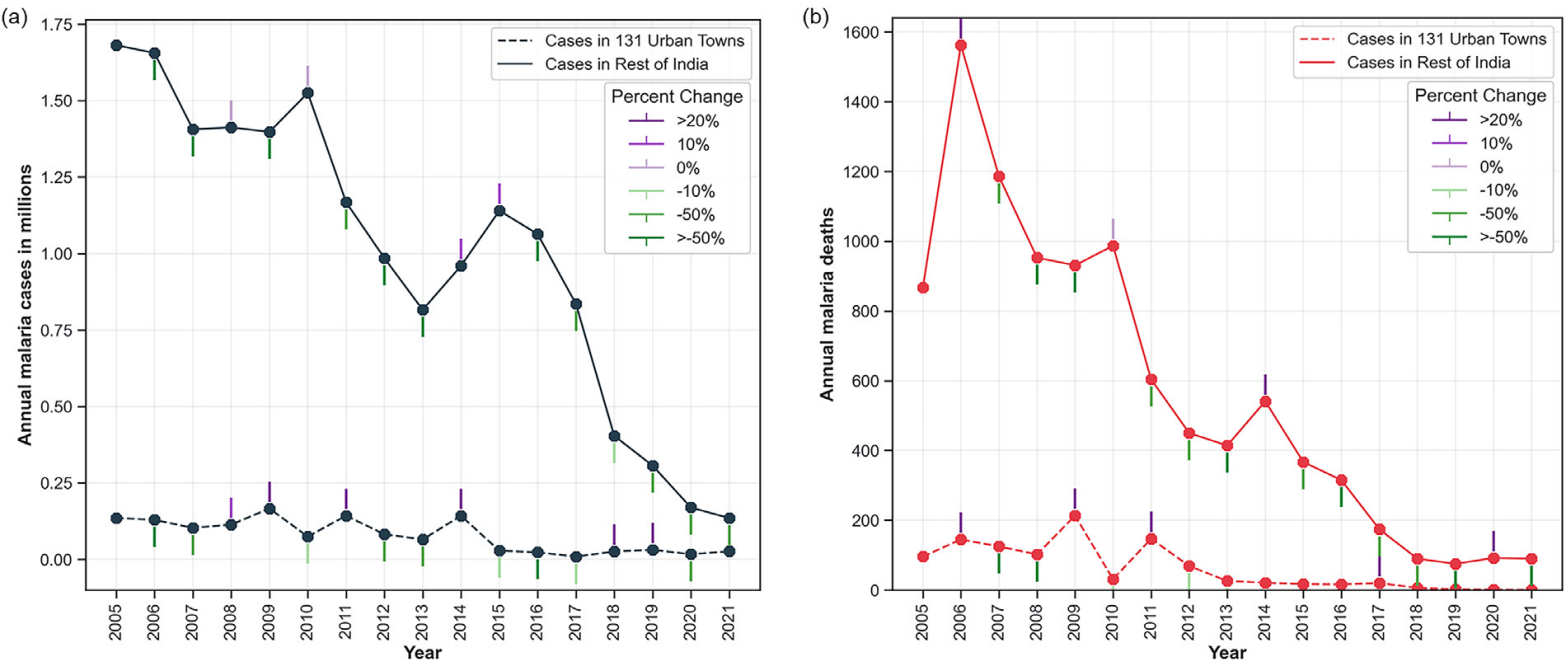
Malaria (a) cases and (b) deaths reported in urban towns and the rest of the country. The bar on each dot represents the percent change observed compared to the previous year. (Data Source: NCVBDC, unpublished).

The comparison of malaria cases and deaths observed in UMS towns and the rest of the country is provided in Figures 3a and 3b, respectively. The case fatality rate observed in urban areas was the highest in 2017 (21.5 per 1000 cases). As shown in Figure 3, urban towns accounted for only 7% of the cases and 10% of the deaths reported. Between 2005 and 2021, the remaining areas contributed to 83-99% of the total cases and 80-100% of the total deaths reported across the country. This suggests that while malaria is widely distributed across the country, it predominantly affects non-urban areas, and a concentric approach is required to stop the transmission in both urban and non-urban regions.

## Recent trends and efforts in malaria control

**Figure 4:**
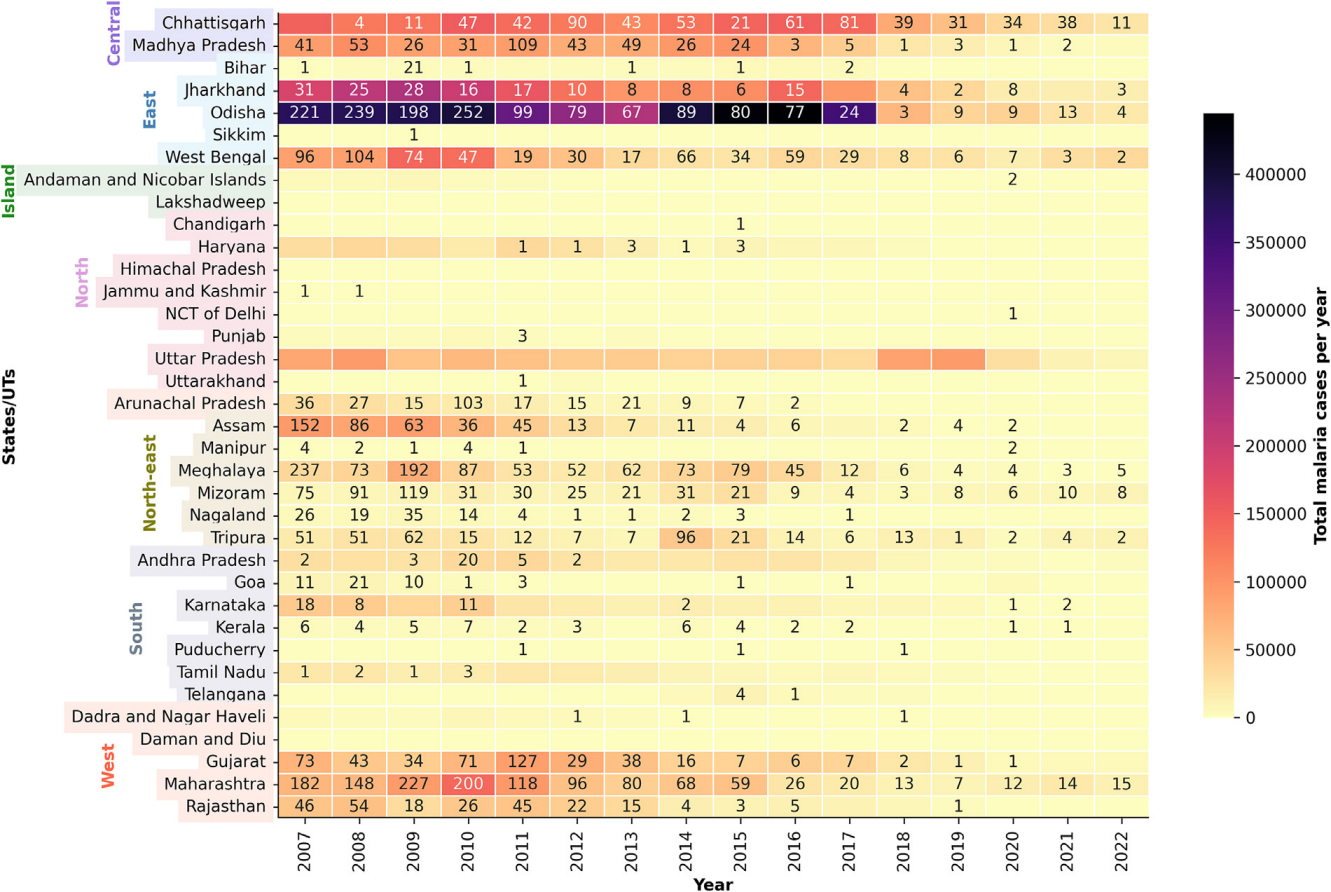
Variations in annual malaria cases and deaths reported in India between 2007 and 2022. The color on each block represents cases; the deaths are represented by numbers (Data source: NCVBDC, unpublished).

The 15-year trend in the annual malaria cases and deaths in the states and UTs between 2007 and 2022 is depicted in Figure 4. The seven N/E states accounted for about 12.7% of the total cases but 37.7% of the deaths reported in India between 2007 and 2010. A drop to 9% of the total cases and 12% of the total deaths was observed between 2011 and 2015. Strategically, malaria control focused on early active and passive case detection and prompt treatment by engaging village link workers to reach inaccessible and remote areas. The introduction of rapid detection tests based on histidine-rich protein-2 (HRP2) aimed at early diagnosis in *P. falciparum* dominant areas [12]. Later, Bivalent RDTs using HRP2 and parasite lactate dehydrogenase were introduced nationwide in 2012 [9,14].

In 2016, the NCVBDC developed the National Framework for Malaria Elimination in India (NFME), aiming to eradicate malaria across the country and enhance overall health and quality of life. The NFME aligns with the World Health Organization's Global Technical Strategy for Malaria 2016-2030 and the Malaria Elimination Roadmap of the Asia Pacific Leaders Malaria Alliance [14]. Due to the high spatial variability in malaria cases across the country that was observed in the past (Figure 4), regional malaria control and the development of necessary health systems have been given due diligence. Under the NFME, states and the UTs were divided into four categories. Category 0 has zero indigenous cases and aims at the prevention of the re-establishment phase. Category 1, or the elimination phase, reports an annual parasite incidence (API) of <1 case per 1000 individuals at risk. Category 2 states have an API of < 1, but few districts in these states have reported an API of >1 and are targeted for pre-elimination. States/UTs with an API of ≥1 have been grouped into category 3 or the intensified control phase [14].

Other important aspects of the NFME are the focus on the districts as the unit for strategy, and planning and implementation, particularly in the high-transmission areas. In 2015, India was a major contributor globally to the *P. vivax* burden, accounting for 47% of the reported cases [16]. Measures ranging from the microscopic level, such as good quality microscopy for detecting all *P. vivax* cases, to conducting large-scale measures, such as operational research for appropriate vector control and introduction of a 14-day radical treatment involving primaquine in infected individuals, became an integral part of malaria control [14].

Our analysis indicates that in the NFME era, Odisha contributed the highest proportion of malaria cases reported in India – ∼41% in 2016 and 2017. This dropped drastically in 2018 (15%) and varied between 11% and 22% in subsequent years. Madhya Pradesh (MP) reported a ∼94% decrease in the total number of cases in 2022 compared to 2016 (Figure 4). In the malaria-prevalent Mandla district of MP, the case counts have steadily declined since 2017, owing to the private sector initiative via the Malaria Elimination Demonstration Project (MEDP). This showcased the significance of public-private collaborations [17]. In contrast, Uttar Pradesh witnessed increased outbreaks in 2018 (20.1%) and 2019 (27.4%) when compared to 2017 (3.8%). The neighboring states of Chhattisgarh and Jharkhand were significantly affected by malaria in the NFME era, with Chhattisgarh accounting for relatively higher proportions (13.6-19.2%) as compared to Jharkhand (8.7-13.9%). West Bengal, Maharashtra, and Tripura have emerged as new hotspots in recent years. West Bengal, in particular, has witnessed a drastic growth, with the highest proportion of cases reported in 2022 (23.7%). Maharashtra contributed 11.9% of cases in 2021, while Tripura contributed 7.8% of the cases in 2022 (Figure 4).

In addition to the NFME, the NCVBDC, with the support of the WHO India country office, implemented the National Strategic Action Plan (NSP) for malaria elimination in India with a concentric focus on the districts. Based on the mean API reported between 2014 and 2016, 678 districts in India were stratified into four categories. The first category focused on the districts and units with no local transmission and case reporting in the previous 3 years. In 2017, the year NSP was launched, 75 (11·1%) districts were classified into category 0, 448 (66%) districts were grouped into category 1; and 46 (6·78%) were classified into category 2. 109 (16·1%) districts that reported API> 2 per 1000 population were grouped into category 3. These districts are earmarked for targeted elimination in the upcoming years. The redistribution of the districts into the categories in the following 3 years after NFME is shown in Figure S1.

Notably, the number of districts grouped under categories 2 and 3 has decreased from 46 (6.8%) and 109 (16.1%) in 2017 to 32 (4.6%) and 10 (1.4%) in 2019, respectively. Accordingly, the proportion of districts in category 0 and category 1 has increased by 138.7% and 10.9%, suggesting remarkable success in reducing malaria prevalence and improving malaria elimination (Figure S1).

**Figure 5:**
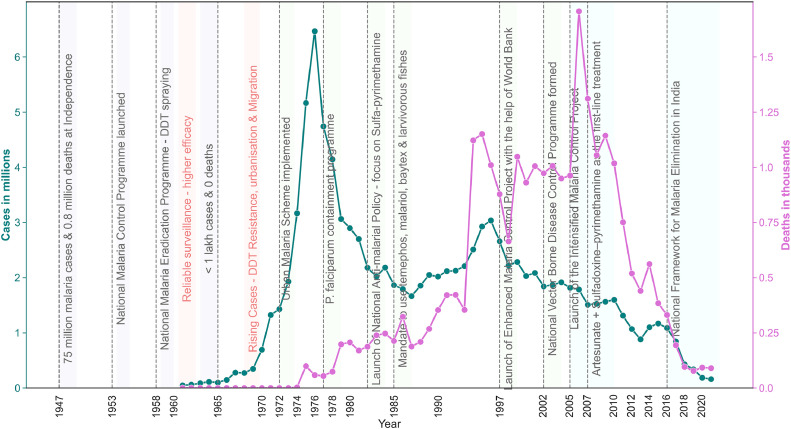
Policy changes in India in response to the malaria cases and deaths reported. (Data source: NCVBDC (unpublished). The data was plotted in Matplotlib version 3.5.2.). DDT, dichlorodiphenyltrichloroethane.

The key policy changes that are discussed above and the annual trends in malaria cases and deaths are summarized in Figure 5.

## The road ahead

India's present focus on malaria control is backed by quantitative case assessment, as different strata have been created based on the annual parasite index. The WHO has provided technical and research support for articulating policies in response to the efficiency of drugs successfully [3] (Text S1.1). The economic stress due to malaria has been estimated to be about $1.9 billion, according to the NCVBDC. Although malaria incidences have decreased over the years, malaria cannot be overlooked even if the API < 1 is achieved. In the past, urbanization, migration, and economic development have been crucial in the spread and resurgence of malaria. India's malaria policy has been historically driven by insecticidal and antimalarial drug resistance. The World Malaria Report, 2022, suggests impending signs of growing resistance to artesunate in combination with sulphadoxine-pyrimethamine, specifically in central India [16].

The COVID-19 pandemic has been the focal point in recent years that has captivated the attention of the government towards the health infrastructure in the country. It has been highlighted that infectious diseases can never be ignored till a near-zero transmission status is achieved; the low socio-economic groups have a higher vulnerability [18]. India distributed 0% of the planned insecticidal-treated nets in 2021 while accounting for 83% of the malaria deaths in the classified Southeast region. Diagnostic testing also decreased by 37 million in 2020 compared to 2019 [16]. Furthermore, malaria, a “local and focal” disease, was significantly impacted by the local ecoclimatic factors that include socio-cultural and behavioral aspects of the residing communities. Along with healthcare seeking and accessibility and treatment compliance, these factors influence the policies enacted by the government. Hence, it is crucial that the impacts of socio-economic, behavioral, and cultural aspects on the intervention strategies are systematically assessed [19].

### Migration, tribal areas, and climate change

The resurgence of infectious diseases due to migration could substantially impact global health security [20]. There are very limited studies in India focusing on migration patterns, as the movements of migrants are difficult to track. At present, malaria is restricted to forested regions, which are home to marginalized 'tribal' populations, and where implementation of intervention strategies focusing on early treatment followed by diagnosis is difficult [3]. Some of these regions are located in inaccessible areas that hinder mitigation measures. For instance, 54.1% of *P. falciparum* cases were reported in Odisha and Chhattisgarh, states with high forest cover and large populations of marginalized communities [21].

The concerning malaria situation in tribal areas, along with the failure to address the needs of ethnic communities, could have adverse consequences on the success of the countrywide efforts aimed at decreasing morbidity and mortality due to malaria. Therefore, these districts need to be prioritized with concerted efforts aiming to adopt high burden to high-impact techniques and holistic socio-economic development at the grassroots level. Moreover, there is a lack of adequate data on the prevalence of malaria based on gender and age. Some studies indicate a higher incidence of malaria in males compared to females [3]. The Government of Odisha initiated a project named DAMaN that targeted high-endemic regions in the state and focused on biennial mass screening followed by treatment and deployment of long-lasting insecticide nets (LLINs) [21]. The DAMaN intervention camps have been effectively linked to a decrease in rural malaria. Based on the efficiency and feasibility of the camps, the Odisha Government extended the DAMaN initiative in 2022 for 5 more years, with the aim of eliminating malaria by 2030 [22]. Similarly, the Malaria-Mukt Bastar Abhiyan, implemented in the Bastar region of Chhattisgarh, was also successful in reducing cases by 50%, from 5272 in 2019 to 2696 cases in 2020 [23]. In the tribal-dominated MP, the Mandla Malaria Elimination Demonstration Project achieved a 91% case reduction with zero indigenous cases by May 2020. These findings suggest that malaria elimination in hard-to-reach, tribal-dominated high-endemic areas is achievable in India [17].

Further, the impending threats of climate change and its influence on the viability of infectious diseases are at the forefront of discussions of the past decade. Back in 2004, the Ministry of Environment and Forest, Government of India, predicted increased outbreaks in high altitude and latitude regions in India and suggested surveillance systems, vector control measures, and the use of public education as remedial measures. The recently published sixth assessment report of the Intergovernmental Panel on Climate Change reports the high likelihood of a significant increase in the health risks attributable to various climate-sensitive diseases. There is very high confidence in the impact scale being determined by adaptation planning, governmental cooperation, health system investments, and emissions control. Therefore, adequate methodological frameworks need to be implemented to assess the true impacts of climate change on malaria in India [24].

### Cross-boundary engagement, private sector involvement, and digital policy

There is a need for cross-boundary engagement to prevent malaria from spreading across international borders [25]. While in vitro studies on the efficacy of the antimalarial drugs, specifically artemisinin derivatives, have reported no decreased sensitivity, clinical resistance has been reported along the Thai-Myanmar and Thai-Cambodia borders [26,27]. The westward spread of this resistance could jeopardize the malaria control program of India, as observed previously when chloroquine resistance was reported. Case management could be a critical factor in the malaria control program as it involves diagnosis, care, and referrals [9].

Additionally, the vision provided by India's Public Health Surveillance for 2035 aims to use the digital revolution happening across the country to align with the citizen-centricity of the National Health Policy 2017 [28,29] and enhance the efforts in disease surveillance through the involvement of the private sector. Niti Aayog, the apex public policy think-tank of the Government of India, suggests an engagement and partnership between public and private healthcare providers to tackle the emerging problems of infectious diseases [28]. According to the estimates from the National Sample Survey conducted in 2014-15, >60% of the patient care was provided by private healthcare facilities [30]. A study in MP reported a significantly higher preference for seeking private healthcare (65%) for malaria treatment when compared to government healthcare [31]. “Paper-based aggregated” surveillance is one of the major reported challenges [32]. The incomplete and inadequate surveillance system in India fails to capture the true burden, which is also affected by the exclusion of the private health sector.

Presently, no well-developed disease reporting systems provide real-time monitoring information to the public, leading to delays in detecting through early warning systems and prompt responses. For an epidemic-prone disease like malaria, delayed reporting along with poor health infrastructure due to the remotely located and inaccessible areas remains a formidable obstacle. Decentralizing the management of the district, revamping the control program, providing financial incentives with better human resources, and integrating other disease-specific control and elimination programs are key measures that would play a critical role in the fight against malaria [32]. The elimination that the government has targeted will be possible if there is a proper mechanism for data dissemination and a good governance framework involving a synergistic multi-stakeholder partnership that will help India strengthen its position in the fight against malaria and avoid any potential resurgence.

Through our policy analysis, we provide a systematic outlook of the intervention strategies implemented in India since independence. However, as much of the efforts continue to be shaped by the reported insecticidal resistance, there is a consensus for methodological research that also accounts for extrinsic factors such as climate change and cross-boundary malaria. Future reviews may include additional interventions targeted in specific states, with a particular emphasis on malaria entomology across the country.

## Declarations of competing interest

The authors have no competing interests to declare.

## Appendix Supplementary materials

Image, application 1
