# Supplementary material for: A historical perspective of malaria policy and control in India
Source: IJID Reg. 2024 Aug 21;12:100428. doi: 10.1016/j.ijregi.2024.100428 (PMC11408002; doi:10.1016/j.ijregi.2024.100428)
Supplement: Supplementary file 1 [file mmc1.docx]

*Supplementary Material*

**A Historical Perspective of Malaria Policy and Control in India**

Avik Kumar Sam^1^, Siddhartha Karmakar^2^, Siuli Mukhopadhyay^2,3^, Harish C. Phuleria^1,3,4, *^

*^5^ Environmental Science and Engineering Department, Indian Institute of Technology Bombay, Mumbai, India*

*^6^ Department of Mathematics, Indian Institute of Technology Bombay, Mumbai, India*

*^7^ Koita Centre for Digital Health, Indian Institute of Technology Bombay, Mumbai, India*

*^8^ Inter-Disciplinary Program in Climate Studies, Indian Institute of Technology Bombay, Mumbai, India*

* Corresponding Author: Harish C. Phuleria; [phuleria@iitb.ac.in](mailto:phuleria@iitb.ac.in)

**Text S1.1. Role of the World Health Organization**

India's remarkable success in reducing malaria can be attributed to a series of commodity and policy interventions and partnerships with WHO. Research support has been provided by WHO in consultation with NCVBDC for articulating policy designs in response to the efficacy of the drugs and treatments. The efficacy of malaria RDTs and bivalent RDTs for the detection of *P. falciparum* cases and artemisinin-based combination therapy (ACT) has been thoroughly examined across the country and even along the international borders shared by India. WHO also provides technical guidance to the NCVBDC on improving the Quality Assurance System on malaria microscopy. For instance, 30 L1 and L2 malaria microscopists have been certified by WHO to provide training for cascading programs in the states that have been executed successfully [[1](#ref1.1)].

The Integrated Health Information Platform (IHIP) initiated by the government, with support from WHO, has enabled real-time monitoring and data reporting, resulting in a better and systematic implementation of the programmatic interventions. The malaria modules presently focus on Odisha and Himachal Pradesh. Information on insecticidal resistance of malaria vectors is also being updated for formulating reformed resistance management plans in the country. WHO has actively endorsed cross-border collaboration among South Asian countries. A strategic framework was jointly drafted by WHO and NCVBDC in 2017, followed by the first Indo-Bhutan meeting organized in 2019 that resulted in an action plan for malaria elimination and has been ratified by districts along the borders. WHO has also focused on "Capacity Building" through the National Strategic Plan that sought to sensitize states and union territories in building resources centered around malaria elimination [[1](#ref1.1),[2](#ref1.2)].

The Roll Back Malaria (RBM) Partnership to End Malaria and WHO have collaborated to launch the "High burden to high impact: A targeted response" (HBHI) that seeks to implement a response plan driven by individual countries for achieving rapid and sustainable malaria impact. The HBHI, implemented across India and ten African countries with high malaria burdens, involves rigid commitment from all stakeholders, high-level political leadership, and country ownership. The HBHI intends to resolve political promises into tangible initiatives, enhance the effective utilization of strategies for achieving significant outcomes, and execute highly effective global policies, strategies, and guidelines [[1](#ref1.1)].

By 2019, India witnessed a significant reduction in malaria cases and was one of the two HBHI countries to achieve this [[2](#ref1.2)]. In association with the NCVBDC and the RBM, WHO implemented holistic measures in the four high-burden states of Jharkhand, MP, Chhattisgarh, and West Bengal. WHO provided intensive support in situation analysis and strategizing state- and district-intensive plans. Training sessions have been organized by both NCVBDC and WHO for officers and consultants engaged in establishing context-specific strategies for mitigating and reducing burden in each district.

**References:**

[1] Kumari R, Kumar A, Dhingra N, Sharma SN. Transition of malaria control to malaria elimination in India. J Commun Dis 2022;54:124–40.

[2] Narain JP, Nath LM. Eliminating malaria in India by 2027: the countdown begins! Indian J Med Res 2018;148:123–6. doi: [10.4103/ijmr.IJMR_1175_18](https://doi.org/10.4103/ijmr.IJMR_1175_18).

**Supplementary Figure**


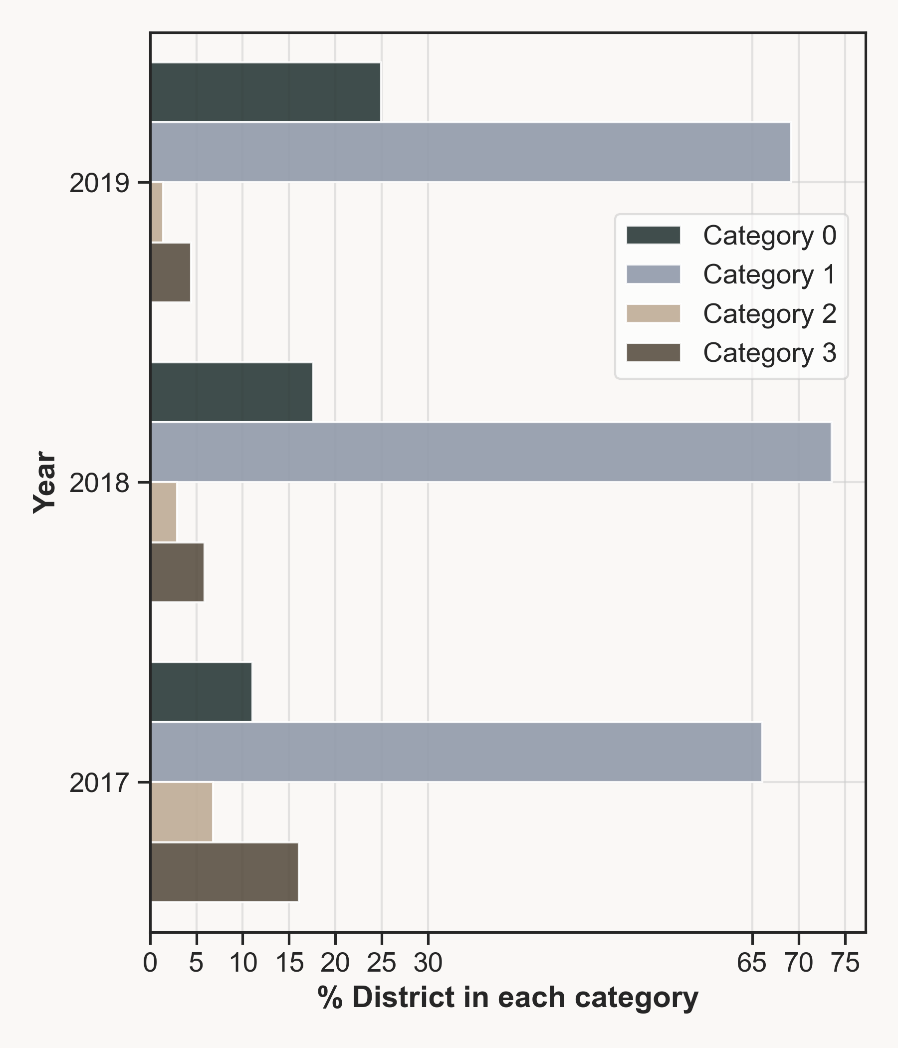


Figure S1. Redistribution of districts under NSP between 2017 and 2019.

(Data source: NCVBDC (unpublished). The data was plotted in Matplotlib version 3.5.2.).
